# Women’s Perspective on Self-Breast Examination

**DOI:** 10.7759/cureus.58962

**Published:** 2024-04-24

**Authors:** Susithra Radhakrishnan, Pankaj B Shah

**Affiliations:** 1 Community Medicine, Sri Ramachandra Institute of Higher Education and Research, Chennai, IND

**Keywords:** breast cancer, breast lump, benefit, barrier, self-breast examination

## Abstract

Background

Breast cancer is the leading cause of cancer death among women in the world. Timely detection is important to reduce the rate of deaths. Among the various screening modalities, self-breast examination is suggested as an easy, inexpensive method, especially in low-resource settings.

Objective

To understand women’s perspective on self-breast examination and analyze the benefits and barriers of self-breast examination.

Method

The total number of study participants was 100. After obtaining informed consent, the study participants were interviewed using a semi-structured questionnaire on their perspective towards self-breast examination along with the benefits and barriers of performing the examination.

Results

Among the study participants, 66% of women were aware of self-breast examination. Only 8% were aware of the age to begin self-breast examination. Lack of privacy was considered as a barrier in 18% of women and embarrassment as a barrier was 14%. Almost all (99%) agreed that completing a self-breast examination each month may help them find breast lumps early.

Conclusion

The participants were reasonably aware of the term self-breast examination. But the clearcut procedure, the age to begin the examination and changes to be picked up on the examination were all unknown and hence must be emphasized at the society level. Overcoming the barriers and accepting the benefits of self-breast examinations are necessary to adopt this examination as a regular practice.

## Introduction

Worldwide, breast cancer is the most common invasive cancer among women. The incidence of breast cancer has increased rapidly in recent years. Among the sustainable development goals (SDGs), goal 3 aims to prevent non-communicable diseases by nearly one-third [1]. In spite of the lower incidence of breast cancer in India when compared to developed nations, the mortality rates are very high owing to the higher proportion of cases presenting only in the advanced stages of the disease [2]. There are plenty of treatment advances, but detecting breast cancer as early as possible is important to maximize the prognosis and the potential for good health outcomes [3]. Many women are still neither aware of breast cancer nor its symptoms. The lack of awareness about the early warning signs and symptoms is the reason for the late presentation to the health care facility. Though various diagnostic methods fail to detect breast cancer at early stages, the self-breast examination will be a very helpful technique to detect early breast cancers. 

Self-breast examination is considered to be a simple, non-expensive, relatively quick, non-invasive, and harmless intervention that can be performed by every woman herself at home [4]. This examination will help women become familiar with their breasts by means of appearance, contour, and consistency and to reach out to the health facility if they find something abnormal, either on inspection or palpation. Most of the women may not be aware of this examination, and even if they are aware, they are neither unsure of the benefits nor the procedure of this examination. Apart from these, there can also be some challenges or difficulties in performing a self-breast examination.

Although there is no solid evidence that instructing women to perform regular self-breast examinations lowers mortality in low-income countries, it is more likely to raise awareness about breast cancer [5]. Hence, this study was focused on understanding the perspective of women towards self-breast examination and also assessing the benefits and barriers of self-breast examination.

## Materials and methods

The study was a cross-sectional study conducted among women 35 years of age and older attending the out-patient department at the rural health training center of Sri Ramachandra Medical College & Research Institute, Tiruvallur district, Chennai, Tamil Nadu, India, during August and September 2022. Based on the review of the literature, about 80% of the participants in the study were capable of self-breast examination after training [6]. Based on this prevalence of 80%, keeping relative precision as 8, the sample size was calculated using the relative precision formula: N = 4pq/d^2^, substituting the values in the formula, 4 × 80 x 20/8 x 8 = 100.

Thus, the sample size was calculated to be 100 using the relative precision formula. So, 100 women attending the outpatient department at the Rural Health Training Center were selected for the study. Women in the age group of 35 years and older were included in the study. Breastfeeding women, women with amastia, and women with symptoms of cyclical mastalgia were excluded from the study. Women with amastia were excluded because they lack breasts, and those with cyclical mastalgia were excluded because they would not be willing to do the examination until the subsidence of symptoms because of discomfort and pain. Data entry was done using Statistical Package for Social Science (SPSS) Software, version 16 (IBM Corp., Armonk, New York, USA).

The study was started after obtaining prior permission from the Institutional Ethics Committee of Sri Ramachandra University of Higher Education and Research (IEC number: CSP-MED/22/AUG/79/128, dated: 03/09/2022). Written informed consent was obtained from the participants, and then the data was collected. The questions in the semi-structured questionnaire were asked one-to-one by the researcher. Every single piece of information collected from the participants was kept confidential.

Data collection

The study was conducted using a semi-structured questionnaire, which consists of the following parts: Part-A: Questions related to sociodemographic characteristics such as age, marital status, socioeconomic class, number of pregnancies, level of education, and employment status. Part-B: Questions related to knowledge and awareness of self-breast examination, mainly focusing on the seriousness of the breast cancer problem, the sex group that gets affected, the types of breast cancer screening methods, the age to begin and the frequency of performing self-breast examination, the procedural idea, and the changes to be noted on this examination. Part-C: Questions about the barriers and benefits of the self-breath examination. Barriers such as inadequate privacy, difficulty remembering every month to perform the procedure, awkwardness or embarrassment, and neglect as a result of various other problems in their lives were determined. Awareness of the benefits, such as the usefulness of this examination for earlier detection, self-care for their breasts, and early detection leading to a decreased rate of death from breast cancer, was also analyzed. The questionnaire in the table form is included in Appendix 1 (Tables 6-8).

Basic descriptive statistical analysis was done using Statistical Package for Social Science (SPSS) software, version 16 (IBM Corp., Armonk, New York, USA). The chi-square test was applied to test the association between the categorical data. The level of significance was set at a p-value of ≤0.05.

## Results

Sociodemographic characteristics

In this study, 60% of the participants were between 35 and 45 years of age. 89% of the study population were married. Nearly half (43%) of them studied beyond middle school. The majority of the population was unemployed (68%) (Table 1).

**Table 1:** Questions related to sociodemographic characteristics.

S. no	Characteristics	Category	Frequency (percentage)
1	Age	35 to 45 years	60 (60%)
		>45 years	40 (40%)
2	Marital status	Married	89 (89%)
		Divorced/widowed/separated	11 (11%)
3	Number of pregnancies	Nullipara	2 (2%)
		One pregnancy	12 (12%)
		Two pregnancies	63 (63%)
		Three pregnancies	19 (19%)
		Four pregnancies	4 (4%)
4	Level of education	Primary school	25 (25%)
		Middle school	32 (32%)
		High school	18 (18%)
		Diploma	13 (13%)
		Degree	12 (12%)
5	Socioeconomic class	Class 1	28 (28%)
		Class 2	15 (15%)
		Class 3	26 (26%)
		Class 4	21 (21%)
		Class 5	10 (10%)
6	Employment status	Employed	32 (32%)
		Unemployed	68 (68%)

Perspective of self-breast examination

In our study, 66% of the participants were aware of the self-breast examination. Though aware, only 31% of women knew that self-breast examination was a screening method for breast cancer. Very few (8%) of them knew the age to begin self-breast examinations (Table 2).

**Table 2:** Questions related to the perspective of self-breast examination. IUCD: intrauterine contraceptive device.

S. no.	Characteristics	Category	Frequency (percentage)
1	Breast cancer is highly prevalent and it is a leading cause of death among women	Disagree	6 (6%)
		Neither agree nor disagree	24 (24%)
		Agree	70 (70%)
2	Which sex group does breast cancer affect?	Female only	65 (65%)
		Both female and male	35 (35%)
3	Does early detection improve the chance of survival?	Yes	97 (97%)
		No	3 (3%)
4	Do you know the types of breast cancer screening methods?	Yes	89 (89%)
		No	11 (11%)
5	Which types of breast cancer screening methods do you know?	Breast self-examination	31 (31%)
		Clinical breast examination	30 (30%)
		Mammography	8 (8%)
		Ultrasound	6 (6%)
		Magnetic resonance imaging	14 (14%)
		None	11 (11%)
6	Do you have a family history of breast cancer?	Yes	4 (4%)
		No	96 (96%)
7	Which contraceptive method do you use?	Oral contraceptive	5 (5%)
		Injectable contraceptive	0 (0%)
		Implants	0 (0%)
		Condom	1 (1%)
		IUCD	13 (13%)
		None	81 (81%)
8	Have you ever heard of a self-breast examination?	Yes	66 (66%)
		No	34 (34%)
9	When should a girl begin a self-breast examination?	Less than 20 years	8 (8%)
		20–30 years	13 (13%)
		30–40 years	21 (21%)
		>40 years	24 (24%)
		Don’t know	34 (34%)
10	How often self-breast examination should be performed?	Weekly	3 (3%)
		Monthly	40 (40%)
		Yearly	23 (23%)
		Don’t know	34 (34%)
11	What do you look for during a self-breast examination?	Breast lump	40 (40%)
		Size of the breast	5 (5%)
		Change in nipple and unusual discharge	20 (20%)
		Change in skin color	1 (1%)
		Don’t know	34 (34%)

Barriers and benefits of self-breast examination

The study reveals that 14% of the participants consider the procedure to be embarrassing to them. Lack of remembrance is considered a barrier by 14% of the participants. Almost all (99%) of them agreed that performing a self-breast examination every month would enable them to find breast lumps early (Table 3).

**Table 3:** Questions pertaining to the barriers and benefits of a self-breast examination.

S. no.	Characteristics	Category	Frequency (percentage)
1	Breast self-examination is embarrassing to me	Disagree	83 (83%)
		Neither agree nor disagree	3 (3%)
		Agree	14 (14%)
2	It is hard to remember to do a self-breast examination	Disagree	82 (82%)
		Neither agree nor disagree	4 (4%)
		Agree	14 (14%)
3	I don’t have enough privacy to do a self-breast examination	Disagree	79 (79%)
		Neither agree nor disagree	3 (3%)
		Agree	18 (18%)
4	I have other problems more important than doing a self-breast examination	Disagree	86 (86%)
		Neither agree nor disagree	1 (1%)
		Agree	13 (13%)
5	Self-breast examination causes no harm	Agree	100 (100%)
6	When I do a self-breast examination, I am doing something to take care of myself	Agree	99 (99%)
		Neither agree nor disagree	1 (1%)
7	Completing a self-breast examination each month may help me find breast lumps early	Agree	99 (99%)
		Neither agree nor disagree	1 (1%)
8	Regular self-breast examination decreases the rate of death from breast cancer	Agree	98 (98%)
		Neither agree nor disagree	2 (2%)

Association of perspectives about the breast cancer screening methods with sociodemographic characteristics

The sociodemographic characteristics, age shows statistically significant association with the knowledge about the breast cancer screening methods in this study (Table 4).

**Table 4:** Association of perspectives about breast cancer screening methods with sociodemographic characteristics.

S. no	Sociodemographic characteristics	Category	Awareness about breast cancer screening methods		Chi-square value (χ²)	p-value
			Aware	Not aware		
			Frequency (percentage)	Frequency (percentage)		
1	Age	35 to 45 years	36 (60.0%)	24 (40.0%)	5.68	0.017
		>45 years	33 (82.5%)	7 (17.5%)		
2	Marital status	Married	62 (69.7%)	27 (30.3%)	0.166	0.683
		Divorced/widowed/separated	7 (63.6%)	4 (36.4%)		
3	Level of education	Until middle school	41 (71.9%)	16 (28.1%)	0.532	0.466
		Beyond middle school	28 (65.1%)	15 (34.9%)		
4	Socioeconomic class	Upper and middle-class	49 (71.0%)	20 (29.0%)	0.422	0.516
		Lower class	20 (64.5%)	11 (35.5%)		
5	Employment status	Employed	19 (59.4%)	13 (40.6%)	2.038	0.153
		Unemployed	50 (73.5%)	18 (26.5%)		

Association of perspectives of self-breast examination with sociodemographic characteristics

Sociodemographic characteristics, employment status shows a statistically significant association with knowledge of self-breast examination (Table 5).

**Table 5:** Association of perspectives of self-breast examination with sociodemographic characteristics.

S. no	Sociodemographic characteristics	Category	Awareness of self-breast examination		Chi-square value (χ²)	p-value
			Aware	Not aware		
			Frequency (percentage)	Frequency (percentage)		
1	Age	35 to 45 years	44 (73.3%)	16 (26.7%)	3.595	0.058
		>45 years	22 (55%)	18 (45%)		
2	Marital status	Married	57 (64%)	32 (36%)	1.378	0.24
		Divorced/widowed/separated	9 (81.8%)	2 (18.2%)		
3	Level of education	Until middle school	36 (63.2%)	21 (36.8%)	0.477	0.49
		Beyond middle school	30 (69.8%)	13 (30.2%)		
4	Socioeconomic class	Upper and middle-class	48 (69.6%)	21 (30.4%)	1.261	0.262
		Lower class	18 (58.1%)	13 (41.9%)		
5	Employment status	Employed	28 (87.5%)	4 (12.5%)	9.694	0.002
		Unemployed	38 (55.9%)	30 (44.1%)		

## Discussion

Sociodemographic characteristics

Out of 100 women, 35 years and above, 60% of the participants were in the age group of 35 to 45 years. The majority of the study population was married (89%). The remaining (11%) were divorced/widowed/separated. Nulliparity is considered a risk factor for breast cancer diagnosed after 40 years of age by Kelsey et al. in a review article from New York published in 1993 [7]. In our study, nulliparous women contributed 2%.

Among the study participants, 57% of women didn’t have a level of education beyond middle school. Evidence from a study conducted in Gondar town, Northwest Ethiopia, by Asmare et al. during the period of April to May 2021 showed that the educational attainment of college level and above has a positive impact on their knowledge and practice of self-breast examination [8]. As far as employment status is concerned, 68% of the study participants were unemployed. Employment status is related to awareness and participation in breast screening modalities in a study performed by Wang et al. from July to November 2016 in Sydney [9].

Perspective of self-breast examination

In our study, 70% of women agreed that breast cancer is highly prevalent and is a leading cause of death among women. A higher proportion of 65% were unaware that it affects both age groups. Few of the participants (11%) don’t know any of the breast cancer screening methods. In our study, 4% of the participants had a family history of breast cancer. A study by Liu et al., performed from patients with breast cancer recruited from March 2014 to July 2017 in the Department of Breast Surgery, Tianjin Medical University Cancer Institute & Hospital, China, revealed that 5-10% of the causes of breast cancer are familial [10]. 

Oral contraceptive use has been associated with increased risk for invasive breast cancer in a prospective cohort study conducted in the United States by Burchardt et al., which included 113,187 women from the Nurses’ Health Study II with recalled information on oral contraceptive usage from 13 years of age to baseline (1989) and updated data on usage until 2009 collected via biennial questionnaires, with a total of 5799 breast cancer cases identified until the end of 2017 [11]. In our study, 5% of them were using oral contraceptives, and they may or may not be considered at risk.

Among the study participants, 66% of them have heard of self-breast examinations. A study performed at Lucknow by Shipra et al. from December 2020 to November 2021 came up with the result that only 7.55% of rural women have heard of self-breast examination, which is comparatively much less when compared to our study [12]. A similar study conducted in the Volta region of Ghana by Dadzi et al. in 2010 revealed that 43.3% of participants had heard of self-breast examinations [13]. So, compared to other studies, our study participants had an increased percentage of being heard during self-breast examination.

In our study, 8% of the participants were conscious that this examination should begin from less than 20 years of age. Similar studies in the Lucknow and Volta regions of Ghana showed that the percentage of women who were aware of the correct age to begin this examination, which is less than 20 years of age, was 0.94% and 47.2%, respectively [12,13]. Surprisingly, 40% of them knew the frequency to perform a self-breast examination. In a similar study conducted in rural coastal Karnataka, only 20.7% knew the correct frequency to perform this examination [14]. Though many of the study participants have heard about this self-breast examination, the knowledge about the age to begin and the frequency of performing the examination was poor, according to our study results.

A fair proportion of about 66% had an idea about even one of the changes to be picked up on the self-breast examination. When asked about the types of breast screening methods, responses were 31% for self-breast examination, 30% for clinical breast examination, 8% for mammography, 6% for ultrasound, and 14% for magnetic resonance imaging. Other similar studies by Al-Ismaili et al. and Abeje et al. also revealed increased knowledge about the types of breast cancer screening methods [15,16].

Barriers and benefits of self-breast examination

When assessing the barriers to self-breast examination, embarrassment and lack of privacy accounted for 14% and 18%, respectively. In a similar study conducted at three selected PHCs out of six centers that provide a variety of primary healthcare services for women of all age groups in Gaza City, Palestine, from March 2017 to June 2017 by Baloushah et al., embarrassment as a barrier was only 0.9%, and privacy issues accounted for only 2.5% [17]. This shows variation in the perceived barriers among women from different countries.

All the participants accepted that the self-breast examination caused no harm. Almost 99% of the participants agreed that completing a self-breast examination every month would help them detect any breast lumps early.

Association of perspectives about the breast cancer screening methods with sociodemographic characteristics

The sociodemographic characteristic, age showed a statistically significant association with awareness of breast cancer screening methods (χ² = 5.680, p = 0.017). Other variables like marital status, level of education, socio-economic class, and employment status didn’t show an association with awareness of breast cancer screening methods.

Association of perspectives of self-breast examination with sociodemographic characteristics

There is a statistically significant association between employment status and the awareness of self-breast examination (χ² = 9.694, p = 0.002). Previous studies in the literature done by Burchardt et al. in the United States in 2017 and Baloushah et al. in the Gaza City of Palestine from March 2017 to June 2017 revealed similar results [11,17].

Sociodemographic characteristics, age did not show a statistically significant association with the awareness of self-breast examination in this study. Similar studies conducted in Oman by Al-Ismaili et al. from January to December 2018 and in Rajasthan (India) by Kumawat et al. from 2019 to 2020 have shown the same results [15,18].

Variables like marital status, level of education, and socioeconomic status also did not show a statistically significant association with the awareness of self-breast examination. As per the Indian national program on NCD, population-based screening is planned for all cancers.

Limitations

Since the study was conducted in a particular center alone, a multi-centric study could have been done to improve the generalizability of the study results. The study could have included the long-term follow-up of the participants on their understanding of the importance of self-breast examination.

## Conclusions

Every woman should understand that self-breast examination is a useful screening tool for the early detection of breast neoplasms. Despite being heard of the term self-breast examination, the age to begin, the frequency to perform, and the changes to be noted by oneself are the knowledge gaps being observed in this study. Overcoming the obstacles to performing and understanding the importance of this examination are the essential changes women can bring up as a part of self-care. Sociodemographic characteristics, age shows a statistically significant association with knowledge about breast cancer screening methods. Employment status shows a statistically significant association with knowledge of self-breast examination. To conclude, performing self-breast examinations on a regular basis will help women to be breast aware.

## Appendices

Appendix 1

**Table 6:** Part-A: Questions related to sociodemographic characteristics.

S. no.	Characteristics	Category
1	Age	---Years
2	Marital status	A. Single, B. Married, C. Divorced/widowed/separated
3	Number of pregnancy	----Pregnancies
4	Level of education of female	A. Primary school, B. Middle school, C. High school, D. Junior college, E. Diploma, F. Degree
5	Monthly family income	----Rupees
6	Socioeconomic class (based on Modified B. G. Prasad Socioeconomic Status Scale, updated for the year 2022)	A. Class 1, B. Class 2, C. Class 3, D. Class 4, E. Class 5
7	Employment status	A. Employed, B. Unemployed

**Table 7:** Part-B: Questions related to knowledge and awareness of self-breast examination. IUCD: intrauterine contraceptive device.

S. no.	Characteristics	Category
1	Breast cancer is highly prevalent and it is a leading cause of death among women	A. Strongly disagree, B. Disagree, C. Neither agree nor disagree, D. Agree, E. Strongly agree
2	Which sex group does breast cancer affect?	A. Female only, B. Male only, C. Both male and female are affected
3	Does early detection improve chance of survival?	A. Yes, B. No
4	Do you know types of breast cancer screening methods?	A. Yes, B. No
5	Which types of breast cancer screening methods do you know?	A. Breast self-examination, B. Clinical breast examination, C. Mammography, D. Ultrasound, E. Magnetic resonance imaging
6	Do you have family history of breast cancer?	A. Yes, B. No
7	Which contraceptive method do you use?	A. Oral contraceptive, B. Injectable contraceptive, C. Implants, D. Condom, E. IUCD, F. Specify if other
8	Have you ever heard self-breast examination?	A. Yes, B. No
9	When should a girl begin self-breast examination?	A. Less than 20 years, B. Above 20 years, C. 20-30 years, D. Specify if others
10	How often self-breast examination should be performed?	A. Weekly, B. Monthly, C. Yearly, D. I don’t know
11	What do you look during self-breast examination?	A. Breast lump, B. Size of the breast, C. Change in nipple and unusual discharge, D. Change in skin color

**Table 8:** Part-C: Questions pertaining to the barriers and benefits in self-breast examination

S. no.	Characteristics	Category
1	Self-breast examination is embarrassing to me	A. Disagree, B. Neither agree nor disagree, C. Agree
2	It is hard to remember to do self-breast examination	A. Disagree, B. Neither agree nor disagree, C. Agree
3	I don’t have enough privacy to do self-breast examination	A. Disagree, B. Neither agree nor disagree, C. Agree
4	I have other problems more important than doing self-breast examination	A. Disagree, B. Neither agree nor disagree, C. Agree
5	Self-breast examination causes no harm	A. Agree, B. Neither agree nor disagree, C. Disagree
6	When I do self-breast examination, I am doing something to take care of myself	A. Agree, B. Neither agree nor disagree, C. Disagree
7	Completing self-breast examination each month may help me find breast lumps early	A. Agree, B. Neither agree nor disagree, C. Disagree
8	Regular self-breast examination decreases the rate of death from breast cancer	A. Agree, B. Neither agree nor disagree, C. Disagree
